# ED Visits for Schizophrenia Spectrum Disorders During the COVID-19 Pandemic at 5 Campus Health Systems

**DOI:** 10.1001/jamanetworkopen.2023.49305

**Published:** 2023-12-27

**Authors:** Parvati Singh, Saira Nawaz, Eric E. Seiber, Ian Bryant, Kyle Moon, Heather Wastler, Nicholas J. Breitborde

**Affiliations:** 1 Division of Epidemiology, College of Public Health, The Ohio State University, Columbus; 2 Center for Health Outcomes and Policy Evaluation Studies, College of Public Health, The Ohio State University, Columbus; 3 Division of Health Services Management and Policy, College of Public Health, The Ohio State University, Columbus; 4 Department of Economics, University of Cincinnati, Cincinnati, Ohio; 5 Early Psychosis Intervention Center, Department of Psychiatry and Behavioral Health, College of Medicine, The Ohio State University, Columbus

## Abstract

**Importance:**

Although substantial research has reported grave population-level psychiatric sequelae of the COVID-19 pandemic, evidence pertaining to temporal changes in schizophrenia spectrum disorders in the US following the pandemic remains limited.

**Objective:**

To examine the monthly patterns of emergency department (ED) visits for schizophrenia spectrum disorders after the onset of the COVID-19 pandemic.

**Design, Setting, and Participants:**

This observational cohort study used time-series analyses to examine whether monthly counts of ED visits for schizophrenia spectrum disorders across 5 University of California (UC) campus health systems increased beyond expected levels during the COVID-19 pandemic. Data included ED visits reported by the 5 UC campuses from 2016 to 2021. Participants included persons who accessed UC Health System EDs had a diagnosis of a psychiatric condition. Data analysis was performed from March to June 2023.

**Exposures:**

The exposures were binary indicators of initial (March to May 2020) and extended (March to December 2020) phases of the COVID-19 pandemic.

**Main Outcomes and Measures:**

The primary outcome was monthly counts of ED visits for schizophrenia spectrum disorders. *International Statistical Classification of Diseases and Related Health Problems, Tenth Revision *diagnosis codes, categorized within Clinical Classification Software groups, were used to identify ED visits for schizophrenia spectrum disorders and all other psychiatric ED visits, from the University of California Health Data Warehouse database, from January 2016 to December 2021. Time-series analyses controlled for autocorrelation, seasonality, and concurrent trends in ED visits for all other psychiatric conditions.

**Results:**

The study data comprised a total of 377 872 psychiatric ED visits, with 37 815 visits for schizophrenia spectrum disorders. The prepandemic monthly mean (SD) number of ED visits for schizophrenia spectrum disorders was 519.9 (38.1), which increased to 558.4 (47.6) following the onset of the COVID-19 pandemic. Results from time series analyses, controlling for monthly counts of ED visits for all other psychiatric conditions, indicated 70.5 additional ED visits (95% CI, 11.7-129.3 additional visits; *P* = .02) for schizophrenia spectrum disorders at 1 month and 74.9 additional visits (95% CI, 24.0-126.0 visits; *P* = .005) at 3 months following the initial phase of the COVID-19 pandemic in California.

**Conclusions and Relevance:**

This study found a 15% increase in ED visits for schizophrenia spectrum disorders within 3 months after the initial phase of the pandemic in California across 5 UC campus health systems, underscoring the importance of social policies related to future emergency preparedness and the need to strengthen mental health care systems.

## Key Points

**Question:**

Was the COVID-19 pandemic associated with increased emergency department (ED) visits for schizophrenia spectrum disorders?

**Findings:**

This cohort study of 377 872 psychiatric ED visits at 5 University of California campus health systems found that ED visits for schizophrenia spectrum disorders increased by approximately 15% following the initial phase of the COVID-19 pandemic.

**Meaning:**

These findings suggest that the COVID-19 pandemic may have differentially impacted ED utilization for severe psychiatric conditions, including schizophrenia spectrum disorders, underscoring the importance of social policies related to future emergency preparedness and the need to strengthen mental health care systems.

## Teaser

This cohort study examines the monthly pattern of emergency department (ED) visits for schizophrenia spectrum disorders during the COVID-19 pandemic across 5 large University of California Health Systems campuses.

## Introduction

The COVID-19 pandemic has brought about noteworthy psychiatric consequences, with multiple studies reporting heightened prevalence of psychological distress, anxiety,^1,2,3^ depression,^1,2,3,4^ substance use,^1,5^ and suicidal ideation.^1^ A number of factors have likely contributed to the psychological toll of the pandemic, including grief from the unexpected death of loved ones,^6^ financial stress from unemployment, reductions in work hours, disruptions in business,^2,4,7,8,9^ and social isolation,^7,8,9^ among others. Risk mitigation measures in response to the pandemic (such as stay-at-home orders, prioritization of acute over long-term or chronic conditions, and increased emergency response to COVID-19 cases) have corresponded with reduced access to psychiatric care, quality, and care delivery.^10^ Extant literature has demonstrated rapid reductions in emergency department (ED) utilization for mental health services over the course of the pandemic,^11,12,13,14,15,16^ but substantial variation in the magnitude of change has been observed.^11,12,15,16^ No changes have been observed for suicide mortality,^17^ but meta-analytic studies^18,19,20,21,22,23,24^ suggest increases in self-reported incidence and mixed evidence of changes in ED visits for suicidal ideation and self-harm, whereas reductions in ED utilization have been reported for mood disorders.^11,12,14,16^ Less understood, however, is how health care service utilization has changed for one of the most severe mental illnesses: schizophrenia spectrum disorders.

Schizophrenia spectrum disorders are typically marked by delusions, hallucinations, disordered thinking, apathy, and cognitive deficits.^25^ Globally, these disorders constitute 1 of the top 15 leading causes of disability.^26^ In the US, the lifetime prevalence of schizophrenia spectrum disorders ranges from 0.3% to 0.6%,^25,27,28,29,30^ but its routine epidemiologic surveillance remains difficult.^31^ ED visits for schizophrenia spectrum disorders may provide a useful alternative to formal psychiatric surveillance, because the treatment for schizophrenia episodes likely begins with emergency services,^32,33^ and EDs serve both uninsured and insured patient populations.^34^ Previous research^35^ points to the utility of ED visits in gauging acute responses to ecological stressors, with evidence of both procyclic and countercyclic trends in psychiatric ED visits in response to macrosocial shocks in the US. The present study extends this approach to determine whether the onset of the COVID-19 pandemic may have exacerbated ED visits for schizophrenia spectrum disorders at the population level.

Large, macrosocial shocks may correspond with increased incidence of psychiatric disorders.^36^ Macrosocial shocks are events or developments that have a major and widespread impact on a society or multiple societies.^36,37,38,39^ These shocks often transcend individual or localized effects and have far-reaching consequences on various aspects of social, economic, and political life. Some examples of macrosocial shocks that may impact psychiatric outcomes include economic recessions, pandemics, natural disasters, wars, and terrorist attacks.^36,37,38,39^ Two leading hypotheses may explain the association of macrosocial shocks with mental health outcomes.^40^ First, the uncovering hypothesis asserts that the increase in psychiatric cases associated with macrosocial shocks are due to (1) increased reporting of disorders, thereby revealing new cases, and/or (2) disruptions in stability (eg, employment or insurance) that exacerbate preexisting conditions.^41,42,43^ Second, the provocation hypothesis posits that increased stress, maladaptive behaviors, fear, anticipation, and uncertainty can lead to or provoke new psychiatric disorders.^40^

In the case of the COVID-19 pandemic, a constellation of interrelated factors may contribute to exacerbations in schizophrenia spectrum disorders: information saturation (ie, the COVID-19 infodemic), increased isolation, increased stress, reduced social support, and interrupted mental health care.^1,6,10,44,45,46^ Interruptions in mental health care due to access issues, restrictions in use of outpatient clinics, and disruptions in transportation may have caused individuals to turn to the ED for nonurgent clinical issues that typically would have been seen in an outpatient setting.^10^ Such shocks may also initiate psychosis, with increased stress triggering inflammatory responses linked to psychosis risk.^47,48,49,50^ In addition, previous reports^45^ have noted an association of coronavirus exposure and/or treatments with psychotic symptoms. Provocation and uncovering mechanisms may explain changes in health services utilization for schizophrenia spectrum disorders over the course of the pandemic, as has been observed in research examining ED visits for schizophrenia and psychosis outside the US.^50^

In the present study, we use monthly data from the University of California (UC) system, comprising 5 UC campuses (Los Angeles, Irvine, Davis, San Francisco, and San Diego, California), to examine changes in ED visits for schizophrenia following the start of COVID-19 pandemic.^51^ Contrary to other reports of decline in ED use for psychiatric emergencies,^11,13,52,53,54^ we hypothesize an increase in ED visits for schizophrenia spectrum disorders.^50^ We use time series analyses and examine whether any detected increase in ED visits for schizophrenia spectrum disorders is associated with the initial acute phase of the COVID-19 pandemic in California (ie, strict pandemic guidelines and stay-at-home orders in March, April, and May 2020) as well as a more extended formulation of the pandemic spanning March to December 2020.

## Methods

### Data and Variables

For this cohort study, we retrieved ED visit data from the University of California Health Data Warehouse (UCHDW), which provides electronic health records–based information on all-payer inclusive visits to ED across the 5 aforementioned UC campuses.^55^ This database was initiated in 2012 and contains data on more than 6 million patients seen at these UC facilities. The UCHDW provides health care data for one of the largest university systems in the US, serving a diverse patient population. UCHDW data have fine temporal resolution and include *International Statistical Classification of Diseases and Related Health Problems, Tenth Revision (ICD-10)* codes, enabling robust, diagnosis-specific, temporal analyses. These data undergo rigorous quality control assessments and are made available to researchers upon request. Details about the UCHDW are available through annual reports published by the Center for Data-driven Insights.^51^

We obtained aggregated, monthly, deidentified data on patient visits to UC EDs per *ICD-10* psychiatric diagnostic groups subsumed within the Clinical Classification Software^56^ from UCHDW for January 2016 to December 2021, for all patients aged 18 years and older. We opted for this categorization because the UCHDW does not provide disaggregated data by specific diagnostic codes owing to concerns of patient identification. We do not include mood disorders with psychotic features within the schizophrenia spectrum disorders categorization owing to diagnosis groups provided within our UCHDW data request. eTable 1 in Supplement 1 presents the list of psychiatric diagnoses groups included in our analyses. Owing to their aggregate nature, our study did not constitute human participants research and was deemed exempt from institutional review board review and the need for informed consent, in accordance with 45 CFR §46. We followed the Strengthening the Reporting of Observational Studies in Epidemiology (STROBE) reporting guidelines for observational cohort studies.

### Statistical Analysis

Data analysis was performed from March to June 2023. We specified our exposure as a binary indicator of the initial phase of the COVID-19 pandemic in California (1 for March-May 2020; 0 otherwise; consistent with research^52^). Our outcome comprised the monthly counts of ED visits for schizophrenia spectrum disorders from January 2016 to December 2021 contained within the UCHDW database. We opted to begin our analytic period starting 2016 to maintain consistent coding of *ICD-10* diagnostic codes over our study period (the transition from *International Classification of Diseases, Ninth Revision* to *ICD-10* occurred in 2015). We examined whether the early phase of the COVID-19 pandemic (March to June 2020; exposure lags of 0, 1, 2, and 3 months) corresponded with an increase in monthly counts of ED visits for schizophrenia spectrum disorders, compared with ED visits for all other (non–schizophrenia spectrum) psychiatric conditions, and compared with the period preceding March 2020 (ie, January 2016 to February 2020). We used the autoregressive integrated moving average (ARIMA) time series methods to account for trend, seasonality, and autocorrelation exhibited by psychiatric ED visits.^57^ ARIMA models are routinely used in examination of temporal shocks to time series data in epidemiology.^58^ The key components of ARIMA models include autoregression, differencing, and moving average.

First, the autoregressive component of ARIMA models captures the relationship between an observation and a specified number of lagged observations. It assumes that the current value of a time series is dependent on its previous values. The autoregressive parameter in ARIMA specifies the order of the autoregressive component and represents the number of lagged observations used for analysis.

Second, the differencing component is used to transform a nonstationary time series into a stationary series. Stationarity refers to the property where the statistical properties of a time series, such as its mean and variance, remain constant over time. Differencing involves subtracting the previous observation from the current one to eliminate trends or seasonality in the data. The differencing parameter in ARIMA represents the order of differencing.

Third, the moving average component models the relationship between the current value of the time series and a linear combination of past error terms. This component smooths out short-term fluctuations in the data and identifies the number of lagged residual errors to include.

The autoregressive, differencing, and moving average terms comprise the ARIMA signature of a time series. This signature provides the counterfactual or uninterrupted patterns of the series in absence of the exposure (in our case, the COVID-19 pandemic). We used iterative sequences per the Box-Jenkins method to determine the ARIMA signature of our outcome series.^59^ Thereafter, we applied our exposure at 0-month to 3-month lags to examine whether the outcome series, adjusted for its ARIMA signature, and controlling for monthly ED visits for all other psychiatric conditions, exhibited higher than expected counts vs the exposure. We also conducted sensitivity tests to gauge whether alternative specification of the binary exposure as 1 for March to December 2020 (0 otherwise) changed our main analytic results. We also examined the change in ED visits for schizophrenia spectrum disorders (without controlling for all other psychiatric ED visits) vs our exposure to give readers a sense of the absolute magnitude of change in our outcome. Exploratory analyses included examination of northern (UC Davis and UC San Francisco) and southern (UC San Diego, UC Irvine, and UC Los Angeles) California campuses separately, to gauge consistency across broad geographic regions within California. Finally, we examined whether the temporal patterning of ED visits for all other psychiatric conditions vs our exposure aligned with expectations of rapid decline following onset of the COVID-19 pandemic from extant research.^52^ We specified 2-tailed tests using statistical significance level of *P* < .05. All analyses were performed using SCA time series software version 6.3 (Scientific Computing Associates).^60^

## Results

Data for this study spanned from 2016 to 2021 (72 months) and included 377 872 psychiatric ED visits, of which 10.0% (37 815) were for schizophrenia spectrum disorders (Table 1). Over the full series, mean (SD) monthly counts of ED visits for schizophrenia spectrum disorders were 525.2 (41.4), with 519.9 (38.1) monthly visits before the pandemic and a marked increase to 558.4 (47.6) visits following March 2020 (Table 1). Conversely, the mean (SD) monthly counts of all other psychiatric ED visits exhibited a decline during the pandemic (4363.0 [396.3] visits) compared with before March 2020 (4723.0 [389.6] visits) (Table 1). eTable 2 in Supplement 1 provides quarterly averages for these visits from January 2020 to December 2021.

**Table 1.** Description of Study Data From University of California Health Data Warehouse, 2016-2021

Reason for ED visit	Total No. of ED visits	Monthly No. of ED visits, mean (SD)		
		Before and during pandemic	Before pandemic	During pandemic
Schizophrenia spectrum disorders	37 815	525.2 (41.4)	519.9 (38.1)	558.4 (47.6)
All other psychiatric conditions	340 057	4723.0 (389.6)	4781.1 (358.9)	4363.0 (396.3)

Abbreviation: ED, emergency department.

The Figure shows the trends in monthly ED visits for schizophrenia spectrum disorders and all other psychiatric conditions across 5 UC campuses from January 2016 to December 2021. Although psychiatric ED visits for non–schizophrenia spectrum disorders (ie, all other psychiatric conditions) declined following March 2020, circled data points show a sharp increase in ED visits for schizophrenia spectrum disorders following the onset of the COVID-19 pandemic. The dotted line across the Figure indicates the upper bound of monthly ED visits for schizophrenia spectrum disorders before March 2020, and we observed a marked increase from this prior upper bound following onset of the COVID-19 pandemic.

**Figure.**
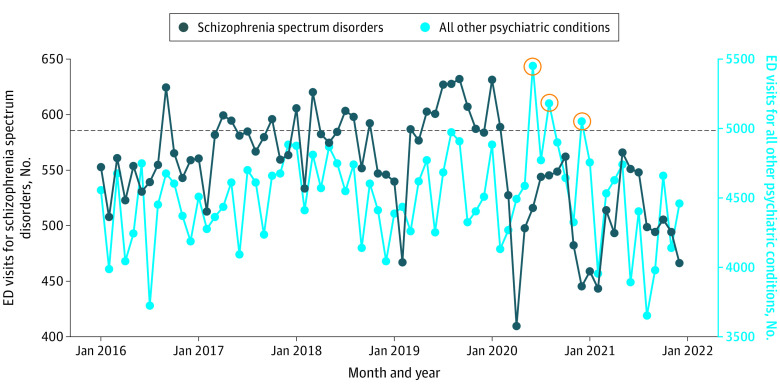
Monthly Frequency of Emergency Department (ED) Visits for Schizophrenia Spectrum Disorders and for All Other Psychiatric Conditions Across 5 University of California Campuses, 2016-2021 Circled observations indicate ED visits for schizophrenia spectrum disorders exceeding their historical upper limit (dashed line) before 2020.

Results from ARIMA analyses appear in Table 2 and Table 3. Application of Box-Jenkins iterative pattern recognition routines, controlling for monthly ED visits for all other psychiatric conditions, identified AR parameter 3 as the ARIMA signature of our outcome series. Application of our binary exposure (March to May 2020) with 0-month to 3-month lags shows an increase in ED visits for schizophrenia spectrum disorders by 70.5 additional visits (95% CI, 11.7-129.3 additional visits; *P* = .02) at exposure lag 1 and by 74.9 additional visits (95% CI, 24.0-126.0 visits; *P* = .005) at exposure lag 3. Robustness checks of ARIMA specifications through examination of autocorrelation function and partial autocorrelation function support our expectation of removal of autocorrelation in our outcome series (eFigure in Supplement 1). Inference from sensitivity tests examining alternative specification of the exposure as March to December 2020 (extended timing of COVID-19 pandemic) aligns with our original test (coefficient at exposure lag 1, 109.4; 95% CI, 45.9-172.1; *P* = .001) (Table 3).

**Table 2.** Time-Series Results Estimating Monthly Counts of Emergency Department Visits for Schizophrenia Spectrum Disorders as a Function of the Initial Phase of the COVID-19 Pandemic (March to May 2020, 0- to 3-Month Lags) From January 2016 to December 2021 Across 5 University of California Campuses

Variable	Coefficient (SE)^a^	*P* value
Constant	352.80 (58.08)	<.001
Emergency department visits for all other psychiatric conditions	0.04 (0.01)	.004
Autoregressive parameter 3	0.27 (0.12)	.03
COVID-19 pandemic (initial phase, March-May 2020)		
Lag 0 mo	−19.5 (26.2)	.46
Lag 1 mo	70.5 (29.7)	.02
Lag 2 mo	−29.5 (29.3)	.39
Lag 3 mo	74.9 (25.8)	.005

^a^ Monthly counts are modeled as a function of the initial phase of the COVID-19 pandemic (binary, March-May 2020, 0-month to 3-month lags), emergency department visits for all other psychiatric conditions, and autocorrelation.

**Table 3.** Time-Series Results Estimating Monthly Counts of Emergency Department Visits for Schizophrenia Spectrum Disorders as a Function of the Extended Phase of the COVID-19 Pandemic (March to December 2020, 0- to 3-Month Lags) From January 2016 to December 2021 Across 5 University of California Campuses

Variable	Coefficient (SE)^a^	*P* value
Constant	293.0 (51.5)	<.001
Emergency department visits for all other psychiatric conditions	0.05 (0.01)	<.001
Autoregressive parameter 12	−0.34 (0.14)	.01
COVID-19 pandemic (extended phase, March-December 2020)		
Lag 0 mo	−20.9 (23.0)	.37
Lag 1 mo	109.4 (32.2)	.001
Lag 2 mo	−43.4 (31.2)	.17
Lag 3 mo	17.6 (22.8)	.44

^a^ Monthly counts are modeled as a function of the extended phase of the COVID-19 pandemic (binary, March-December 2020, 0-month to 3-month lags), emergency department visits for all other psychiatric conditions, and autocorrelation.

Results from robustness checks using an alternate formulation of the exposure with months June, July, and August 2020 coded as 1 (0 otherwise), in keeping with the exposure lags of 1, 2, and 3 months from our main analyses, support our original inference (eTable 3 in Supplement 1). Exploratory analyses by northern and southern California campuses yielded results consistent with a marked increase in ED visits for schizophrenia spectrum disorders in the period of June to August 2020, and we noted a greater response in southern California UC campuses (eTable 4 in Supplement 1) vs UC campuses in northern California (eTable 5 in Supplement 1). Taken together, our analyses indicate there was a mean (SE) of 81.0 (20.6) excess ED visits for schizophrenia spectrum disorders in the 3-month period after the initial phase of the COVID-19 pandemic in California. Given the prepandemic monthly mean (SD) of 519.9 (38.1) ED visits for schizophrenia spectrum disorders in our data, our estimates indicate a 15% increase in these ED visits within 3 months immediately following the initial phase of COVID-19 pandemic (range, 9.6%-25.0%, based on the respective SDs of numerator and denominator).

Examination of the absolute change in ED visits for schizophrenia spectrum disorders (not controlling for ED visits for all other psychiatric conditions) cohere with our main inference in that we observed a mean (SE) increase of 69.5 (27.7) visits at exposure lag 3 (eTable 6 in Supplement 1) when the exposure was modeled as the initial phase of the pandemic, and at exposure lag 1 by 84.8 (37.1) visits, when the exposure was modeled as the extended phase of the COVID-19 pandemic (eTable 7 in Supplement 1). Finally, our examination of monthly ED visits for all other psychiatric conditions indicated a decrease in these visits in response to the exposure, in alignment with extant literature^52^ (eTables 8 and 9 in Supplement 1).

## Discussion

The onset of the COVID-19 pandemic raised concerns of a psychiatric pandemic owing to large-scale disruptions in health care, social isolation, economic uncertainty, and heightened anxiety in the population. This expectation of a potentially lagged increase in severe psychiatric conditions, such as schizophrenia spectrum disorders, following initial phases of the pandemic, is supported by evidence from other countries^50^ but remains underexplored in the US. In this cohort study, we examined whether the expected psychiatric pandemic manifested through increased ED utilization for schizophrenia spectrum disorders across 5 large University of California campus health care systems in the US. Results from our time-series analyses of monthly ED visits counts from 2016 to 2021 suggest a 15% increase in these visits within 3 months following the first phase of the COVID-19 pandemic in California (March-May 2020). We also observed a simultaneous inverse pattern (following pandemic onset) with respect to ED visits for all other psychiatric conditions. Sensitivity tests using alternative, extended formulation of the pandemic (March-December 2020) cohere with our main findings, with 109.4 additional ED visits for schizophrenia spectrum disorders 1 month following exposure.

Concerns about the impact of COVID-19 on population mental health,^6,8^ and schizophrenia spectrum disorders, in particular,^45,61^ were expressed in the pandemic’s earliest months. To our knowledge, however, this study represents the first large-scale empirical evidence of potential exacerbation of schizophrenia spectrum disorders in the US over the course of the COVID-19 pandemic, shown by marked increases in ED visits for this diagnostic group after the pandemic’s onset. Importantly, we account for ED visits for all other psychiatric conditions, and the exposure (pandemic onset) precedes the outcome (ED visit for schizophrenia spectrum disorders). Moreover, our use of time series methods addresses potential confounding by seasonality, temporal trends, and autocorrelation underlying variation in ED visits for schizophrenia spectrum disorders.

In the US, the sequelae of the COVID-19 pandemic were more severe for Black populations.^62,63,64^ Black individuals also appear overrepresented among epidemiologic estimates of schizophrenia diagnosis in the US, potentially stemming from racialized diagnostic criteria and clinician bias, as noted by several scholars.^65,66,67,68,69,70,71^ This circumstance, combined with civil unrest following the police killing of George Floyd in May 2020, may have uniquely impacted ED visits for schizophrenia spectrum disorders among Black populations.^70,71^ Although the examination of this phenomenon remains outside the scope of the present study, we encourage future research to examine racial disparities in schizophrenia spectrum disorders following the COVID-19 pandemic in the US. Increases in schizophrenia cases are likely to exert strain on community mental health centers,^61^ which face substantial challenges in financing and sustaining coordinated specialty care for schizophrenia spectrum disorders,^72,73^ warranting a need for policy interventions to strengthen mental health care systems.

### Limitations

Limitations include that UCHDW data used in this study exclude patients younger than 18 years; thus, any ED visits among this population are not captured in our analyses. It is plausible that many youth with previously undiagnosed psychiatric disorders visited the ED for symptoms of schizophrenia or psychosis-related services during the pandemic, as the uncovering hypothesis would suggest.^41,42,43^ Further research is warranted to determine whether similar trends are observed among younger patient populations. We also did not conduct age and sex-disaggregated analyses (owing to UCHDW data restrictions), and future research may examine whether trends reported in the present study vary by age or sex. One of the highest risk groups for ED use for schizophrenia spectrum disorders comprises persons with unstable housing and unhoused populations. We did not have complete information on housing status for ED visits reported in the UCHDW, and we encourage future researchers to examine the association of the COVID-19 pandemic with psychiatric ED visits in this vulnerable group.

We are also unable to rule out the possibility of potential misclassification of the reason for ED visits following the COVID-19 pandemic. For our inference to be confounded by this differential misclassification, the COVID-19 pandemic would have preceded changes in clinical diagnostic patterns that resulted in overdiagnosis of some ED visits as schizophrenia and/or psychosis and simultaneous underdiagnosis of ED visits for other psychiatric conditions. We encourage future research to examine the robustness of our results in relation to diagnosis misclassification.

Our study is limited to 5 large university campuses within the state of California and, as such, has strong internal validity but limited generalizability. An additional limitation of the present study lies in its inability to identify patients with newly diagnosed schizophrenia. Although a clear increase in ED visits for schizophrenia spectrum disorders was observed, this could be the result of (1) worsened health of existing patients, (2) patients with new diagnoses or those experiencing first episode psychosis, or (3) a combination of new and existing patients. Future studies may compare trends in psychosis-related ED admissions between patients with new and existing schizophrenia using linked data that permit tracking of patient history. Furthermore, owing to UCHDW’s restrictions pertaining to data release of small cell sizes (monthly patient counts <10), we are unable to account for ED visits for schizophrenia spectrum disorders among persons with COVID-19. Prior studies^50,74^ indicate that COVID-19 infections may induce psychosis in some individuals, and this comorbidity may underlie the increase in ED visits for schizophrenia spectrum disorders observed in our analysis. We encourage future researchers to examine this potential comorbidity using larger data sets that provide higher monthly patient counts for these diagnoses.

## Conclusion

This study provides evidence of potential exacerbation of schizophrenia spectrum disorders engendered by the COVID-19 pandemic. The COVID-19 pandemic draws attention to the vulnerability of patients with schizophrenia to macrosocial shocks, underscoring the importance of social policies related to income support, housing, and health insurance for future emergency preparedness and the need to strengthen mental health care systems.

## Notes

Supplement 1. **eTable 1.** Diagnosis Code Groups for Psychiatric-Related ED Visits, per Clinical Classification System (CCS), Retrieved From UCHDW **eTable 2.** Description of Study Data From UCHDW in 3-Month Groups, 2020 to Dec 2021 **eFigure.** Time-Series Graph of Autocorrelation Function (ACF) (Panel A) and Partial Autocorrelation Function (PACF) (Panel B) for ED Visits for Schizophrenia Spectrum Disorders Across 5 UC Campuses (Shown for First 24 Months) **eTable 3.** Time-Series Results Predicting Monthly Values of ED Visits for Schizophrenia Spectrum Disorders From January 2016 to December 2021 Across 5 UC Campuses **eTable 4.** Time-Series Results Predicting Monthly Values of ED Visits for Schizophrenia Spectrum Disorders From January 2016 to December 2021 Across 2 UC Campuses in Northern California (UC Davis, UC San Francisco) **eTable 5.** Time-Series Results Predicting Monthly Values of ED Visits for Schizophrenia Spectrum Disorders From January 2016 to December 2021 Across 3 UC Campuses in Southern California (UC Los Angeles, UC Irvine, UC San Diego) **eTable 6.** Time-Series Results Predicting Monthly Values of ED Visits for Schizophrenia Spectrum Disorders From January 2016 to December 2021 in 5 UC Campuses **eTable 7.** Time-Series Results Predicting Monthly Values of ED Visits for Schizophrenia Spectrum Disorders From January 2016 to December 2021 in 5 UC Campuses **eTable 8.** Time-Series Results Predicting Monthly Values of ED Visits for All Other Psychiatric Conditions From January 2016 to December 2021 in 5 UC Campuses **eTable 9.** Time-Series Results Predicting Monthly Values of ED Visits for All Other Psychiatric Conditions From January 2016 to December 2021 in 5 UC Campuses

Supplement 2.
Data Sharing Statement
