# Error in Figure

**DOI:** 10.1001/jamanetworkopen.2023.56751

**Published:** 2024-01-29

In the Original Investigation titled “ED Visits for Schizophrenia Spectrum Disorders During the COVID-19 Pandemic at 5 Campus Health Systems,”^1^ published December 27, 2023, there was an error in the Figure; the colors for schizophrenia spectrum disorders and all other psychiatric conditions were reversed. This article has been corrected.^1^
