# Supplementary material for: ED Visits for Schizophrenia Spectrum Disorders During the COVID-19 Pandemic at 5 Campus Health Systems
Source: JAMA Netw Open. 2023 Dec 27;6(12):e2349305. doi: 10.1001/jamanetworkopen.2023.49305 (PMC10753394; doi:10.1001/jamanetworkopen.2023.49305)
Supplement: Supplement 1. — eTable 1. Diagnosis Code Groups for Psychiatric-Related ED Visits, per Clinical Classification System (CCS), Retrieved From UCHDW eTable 2. Description of Study Data From UCHDW in 3-Month Groups, 2020 to Dec 2021 eFigure. Time-Series Graph of Autocorrelation Function (ACF) (Panel A) and Partial Autocorrelation Function (PACF) (Panel B) for ED Visits for Schizophrenia Spectrum Disorders Across 5 UC Campuses (Shown for First 24 Months) eTable 3. Time-Series Results Predicting Monthly Values of ED Visits for Schizophrenia Spectrum Disorders From January 2016 to December 2021 Across 5 UC Campuses eTable 4. Time-Series Results Predicting Monthly Values of ED Visits for Schizophrenia Spectrum Disorders From January 2016 to December 2021 Across 2 UC Campuses in Northern California (UC Davis, UC San Francisco) eTable 5. Time-Series Results Predicting Monthly Values of ED Visits for Schizophrenia Spectrum Disorders From January 2016 to December 2021 Across 3 UC Campuses in Southern California (UC Los Angeles, UC Irvine, UC San Diego) eTable 6. Time-Series Results Predicting Monthly Values of ED Visits for Schizophrenia Spectrum Disorders From January 2016 to December 2021 in 5 UC Campuses eTable 7. Time-Series Results Predicting Monthly Values of ED Visits for Schizophrenia Spectrum Disorders From January 2016 to December 2021 in 5 UC Campuses eTable 8. Time-Series Results Predicting Monthly Values of ED Visits for All Other Psychiatric Conditions From January 2016 to December 2021 in 5 UC Campuses eTable 9. Time-Series Results Predicting Monthly Values of ED Visits for All Other Psychiatric Conditions From January 2016 to December 2021 in 5 UC Campuses [file jamanetwopen-e2349305-s001.pdf]

Supplemental Online Content

Singh P, Nawaz S, Seiber EE, et al. ED visits for schizophrenia spectrum disorders during the COVID-19 pandemic at 5 campus health systems. *JAMA Netw Open*. 2023;6(12):e2349305. doi:10.1001/jamanetworkopen.2023.49305

eTable 1. Diagnosis Code Groups for Psychiatric-Related ED Visits, per Clinical Classification System (CCS), Retrieved From UCHDW
eTable 2. Description of Study Data From UCHDW in 3-Month Groups, 2020 to Dec 2021
eFigure. Time-Series Graph of Autocorrelation Function (ACF) (Panel A) and Partial Autocorrelation Function (PACF) (Panel B) for ED Visits for Schizophrenia Spectrum Disorders Across 5 UC Campuses (Shown for First 24 Months)
eTable 3. Time-Series Results Predicting Monthly Values of ED Visits for Schizophrenia Spectrum Disorders From January 2016 to December 2021 Across 5 UC Campuses
eTable 4. Time-Series Results Predicting Monthly Values of ED Visits for Schizophrenia Spectrum Disorders From January 2016 to December 2021 Across 2 UC Campuses in Northern California (UC Davis, UC San Francisco)
eTable 5. Time-Series Results Predicting Monthly Values of ED Visits for Schizophrenia Spectrum Disorders From January 2016 to December 2021 Across 3 UC Campuses in Southern California (UC Los Angeles, UC Irvine, UC San Diego)
eTable 6. Time-Series Results Predicting Monthly Values of ED Visits for Schizophrenia Spectrum Disorders From January 2016 to December 2021 in 5 UC Campuses
eTable 7. Time-Series Results Predicting Monthly Values of ED Visits for Schizophrenia Spectrum Disorders From January 2016 to December 2021 in 5 UC Campuses
eTable 8. Time-Series Results Predicting Monthly Values of ED Visits for All Other Psychiatric Conditions From January 2016 to December 2021 in 5 UC Campuses
eTable 9. Time-Series Results Predicting Monthly Values of ED Visits for All Other Psychiatric Conditions From January 2016 to December 2021 in 5 UC Campuses

This supplemental material has been provided by the authors to give readers additional information about their work.

© 2023 Singh P et al. *JAMA Network Open.*

eTable 1: Diagnosis Code Groups for Psychiatric-Related ED Visits, per Clinical Classification System (CCS), Retrieved From UCHDW

**Psychiatric disorder group (CCS categories)**	**ICD10 codes**
Anxiety Disorders (651)	F064 F4000 F4001 F4002 F4010 F4011 F40210 F40218 F40220 F40228 F40230 F40231 F40232 F40233 F40240 F40241 F40242 F40243 F40248 F40290 F40291 F40298 F408 F409 F410 F411 F413 F418 F419 F42 F422 F423 F424 F428 F429 F430 F4310 F4311 F4312 F488 F489 R452 R453 R454 R455 R456 R457 R4581 R4582 R4583 R4584
Mood Disorders (657)	F0630 F0631 F0632 F0633 F0634 F3010 F3011 F3012 F3013 F302 F303 F304 F308 F309 F310 F3110 F3111 F3112 F3113 F312 F3130 F3131 F3132 F314 F315 F3160 F3161 F3162 F3163 F3164 F3170 F3171 F3172 F3173 F3174 F3175 F3176 F3177 F3178 F3181 F3189 F319 F320 F321 F322 F323 F324 F325 F328 F3281 F3289 F329 F330 F331 F332 F333 F3340 F3341 F3342 F338 F339 F340 F341 F348 F3481 F3489F349 F39
Schizophrenia/psychoses (659)	F060 F062 F200 F201 F202 F203 F205 F2081 F2089 F209 F21 F22 F23 F24 F250 F251 F258 F259 F28 F29
Alcohol and Substance-related disorders (660, 661)	F1010 F1011 F10120 F10121 F10129 F1014 F10150 F10151 F10159 F10180 F10181 F10182 F10188 F1019 F1020 F1021 F10220 F10221 F10229 F10230 F10231 F10232 F10239 F1024 F10250 F10251 F10259 F1026 F1027 F10280 F10281 F10282 F10288 F1029 F10920 F10921 F10929 F1094 F10950 F10951 F10959 F1096 F1097 F10980 F10981 F10982 F10988 F1099 G621 I426 K2920 K2921 K700 K7010 K7011 K702 K7030 K7031 K7040 K709 O99310 O99311 O99312 O99313 O99314 O99315 P043 Q860 F1110 F1111 F11120 F11121 F11122 F11129 F1114 F11150 F11151 F11159 F11181 F11182 F11188 F1119 F1120 F1121 F11220 F11221 F11222 F11229 F1123 F1124 F11250 F11251 F11259 F11281 F11282 F11288 F1129 F1190 F11920 F11921 F11922 F11929 F1193 F1194 F11950 F11951 F11959 F11981 F11982 F11988 F1199 F1210 F1211 F12120 F12121 F12122 F12129 F12150 F12151 F12159 F12180 F12188 F1219 F1220 F1221 F12220 F12221 F12222 F12229 F12250 F12251 F12259 F12280 F12288 F1229 F1290 F12920 F12921 F12922 F12929 F12950 F12951 F12959 F12980 F12988 F1299 F1310 F1311 F13120 F13121 F13129

© 2023 Singh P et al. *JAMA Network Open.*

Psychiatric disorder group (CCS categories)	ICD10 codes
	F1314 F13150 F13151 F13159 F13180 F13181 F13182 F13188 F1319 F1320 F1321 F13220 F13221 F13229 F13230 F13231 F13232 F13239 F1324 F13250 F13251 F13259 F1326 F1327 F13280 F13281 F13282 F13288 F1329 F1390 F13920 F13921 F13929 F13930 F13931 F13932 F13939 F1394 F13950 F13951 F13959 F1396 F1397 F13980 F13981 F13982 F13988 F1399 F1410 F1411 F14120 F14121 F14122 F14129 F1414 F14150 F14151 F14159 F14180 F14181 F14182 F14188 F1419 F1420 F1421 F14220 F14221 F14222 F14229 F1423 F1424 F14250 F14251 F14259 F14280 F14281 F14282 F14288 F1429 F1490 F14920 F14921 F14922 F14929 F1494 F14950 F14951 F14959 F14980 F14981 F14982 F14988 F1499 F1510 F1511 F15120 F15121 F15122 F15129 F1514 F15150 F15151 F15159 F15180 F15181 F15182 F15188 F1519 F1520 F1521 F15220 F15221 F15222 F15229 F1523 F1524 F15250 F15251 F15259 F15280 F15281 F15282 F15288 F1529 F1590 F15920 F15921 F15922 F15929 F1593 F1594 F15950 F15951 F15959 F15980 F15981 F15982 F15988 F1599 F1610 F1611 F16120 F16121 F16122 F16129 F1614 F16150 F16151 F16159 F16180 F16183 F16188 F1619 F1620 F1621 F16220 F16221 F16229 F1624 F16250 F16251 F16259 F16280 F16283 F16288 F1629 F1690 F16920 F16921 F16929 F1694 F16950 F16951 F16959 F16980 F16983 F16988 F1699 F17200 F17201 F17203 F17208 F17209 F17210 F17211 F17213 F17218 F17219 F17220 F17221 F17223 F17228 F17229 F17290 F17291 F17293 F17298 F17299 F1810 F1811 F18120 F18121 F18129 F1814 F18150 F18151 F18159 F1817 F18180 F18188 F1819 F1820 F1821 F18220 F18221 F18229 F1824 F18250 F18251 F18259 F1827 F18280 F18288 F1829 F1890 F18920 F18921 F18929 F1894 F18950 F18951 F18959 F1897 F18980 F18988 F1899 F1910 F1911 F19120 F19121 F19122 F19129 F1914 F19150 F19151 F19159 F1916 F1917 F19180 F19181 F19182 F19188 F1919 F1920 F1921 F19220 F19221 F19222 F19229 F19230 F19231 F19232 F19239 F1924 F19250 F19251 F19259 F1926 F1927 F19280 F19281 F19282 F19288 F1929 F1990 F19920 F19921 F19922 F19929 F19930 F19931 F19932 F19939 F1994 F19950 F19951 F19959 F1996 F1997 F19980 F19981 F19982 F19988 F1999 F550 F551 F552 F553 F554 F558 O355XX0 O355XX1 O355XX2 O355XX3 O355XX4

© 2023 Singh P et al. *JAMA Network Open.*

Psychiatric disorder group (CCS categories)	ICD10 codes
	O355XX5 O355XX9 O99320 O99321 O99322 O99323 O99324 O99325 P0441 P0449 P961 P962 T400X1A T400X1D T400X1S T400X3A T400X3D T400X3S T400X4A T400X4D T400X4S T400X5A T400X5D T400X5S T400X6A T400X6D T400X6S T401X1A T401X1D T401X1S T401X3A T401X3D T401X3S T401X4A T401X4D T401X4S T405X1A T405X1D T405X1S T405X3A T405X3D T405X3S T405X4A T405X4D T405X4S T405X5A T405X5D T405X5S T405X6A T405X6D T405X6S T407X1A T407X1D T407X1S T407X3A T407X3D T407X3S T407X4A T407X4D T407X4S T407X5A T407X5D T407X5S T407X6A T407X6D T407X6S T408X1A T408X1D T408X1S T408X3A T408X3D T408X3S T408X4A T408X4D T408X4S T40901A T40901D T40901S T40903A T40903D T40903S T40904A T40904D T40904S T40905A T40905D T40905S T40906A T40906D T40906S T40991A T40991D T40991S T40993A T40993D T40993S T40994A T40994D T40994S T40995A T40995D T40995S T40996A T40996D T40996S F1223 F1293
Suicide and intentional self-inflicted injury (662)	R45851 T1491 T1491XA T1491XD T1491XS T360X2A T360X2D T360X2S T361X2A T361X2D T361X2S T362X2A T362X2D T362X2S T363X2A T363X2D T363X2S T364X2A T364X2D T364X2S T365X2A T365X2D T365X2S T366X2A T366X2D T366X2S T367X2A T367X2D T367X2S T368X2A T368X2D T368X2S T3692XA T3692XD T3692XS T370X2A T370X2D T370X2S T371X2A T371X2D T371X2S T372X2A T372X2D T372X2S T373X2A T373X2D T373X2S T374X2A T374X2D T374X2S T375X2A T375X2D T375X2S T378X2A T378X2D T378X2S T3792XA T3792XD T3792XS T380X2A T380X2D T380X2S T381X2A T381X2D T381X2S T382X2A T382X2D T382X2S T383X2A T383X2D T383X2S T384X2A T384X2D T384X2S T385X2A T385X2D T385X2S T386X2A T386X2D T386X2S T387X2A T387X2D T387X2S T38802A T38802D T38802S T38812A T38812D T38812S T38892A T38892D T38892S T38902A T38902D T38902S T38992A T38992D T38992S T39012A T39012D T39012S T39092A T39092D T39092S T391X2A T391X2D T391X2S T392X2A T392X2D T392X2S T39312A T39312D T39312S T39392A T39392D T39392S T394X2A T394X2D T394X2S T398X2A T398X2D T398X2S T3992XA T3992XD T3992XS T400X2A T400X2D T400X2S T401X2A T401X2D T401X2S T402X2A T402X2D T402X2S T403X2A T403X2D T403X2S

© 2023 Singh P et al. *JAMA Network Open.*

Psychiatric disorder group (CCS categories)	ICD10 codes
	T404X2A T404X2D T404X2S T405X2A T405X2D T405X2S T40602A T40602D T40602S T40692A T40692D T40692S T407X2A T407X2D T407X2S T408X2A T408X2D T408X2S T40902A T40902D T40902S T40992A T40992D T40992S T410X2A T410X2D T410X2S T411X2A T411X2D T411X2S T41202A T41202D T41202S T41292A T41292D T41292S T413X2A T413X2D T413X2S T4142XA T4142XD T4142XS T415X2A T415X2D T415X2S T420X2A T420X2D T420X2S T421X2A T421X2D T421X2S T422X2A T422X2D T422X2S T423X2A T423X2D T423X2S T424X2A T424X2D T424X2S T425X2A T425X2D T425X2S T426X2A T426X2D T426X2S T4272XA T4272XD T4272XS T428X2A T428X2D T428X2S T43012A T43012D T43012S T43022A T43022D T43022S T431X2A T431X2D T431X2S T43202A T43202D T43202S T43212A T43212D T43212S T43222A T43222D T43222S T43292A T43292D T43292S T433X2A T433X2D T433X2S T434X2A T434X2D T434X2S T43502A T43502D T43502S T43592A T43592D T43592S T43602A T43602D T43602S T43612A T43612D T43612S T43622A T43622D T43622S T43632A T43632D T43632S T43692A T43692D T43692S T438X2A T438X2D T438X2S T4392XA T4392XD T4392XS T440X2A T440X2D T440X2S T441X2A T441X2D T441X2S T442X2A T442X2D T442X2S T443X2A T443X2D T443X2S T444X2A T444X2D T444X2S T445X2A T445X2D T445X2S T446X2A T446X2D T446X2S T447X2A T447X2D T447X2S T448X2A T448X2D T448X2S T44902A T44902D T44902S T44992A T44992D T44992S T450X2A T450X2D T450X2S T451X2A T451X2D T451X2S T452X2A T452X2D T452X2S T453X2A T453X2D T453X2S T454X2A T454X2D T454X2S T45512A T45512D T45512S T45522A T45522D T45522S T45602A T45602D T45602S T45612A T45612D T45612S T45622A T45622D T45622S T45692A T45692D T45692S T457X2A T457X2D T457X2S T458X2A T458X2D T458X2S T4592XA T4592XD T4592XS T460X2A T460X2D T460X2S T461X2A T461X2D T461X2S T462X2A T462X2D T462X2S T463X2A T463X2D T463X2S T464X2A T464X2D T464X2S T465X2A T465X2D T465X2S T466X2A T466X2D T466X2S T467X2A T467X2D T467X2S T468X2A T468X2D T468X2S T46902A T46902D T46902S T46992A T46992D T46992S T470X2A T470X2D T470X2S T471X2A T471X2D T471X2S T472X2A T472X2D T472X2S T473X2A T473X2D T473X2S T474X2A T474X2D

© 2023 Singh P et al. *JAMA Network Open.*

Psychiatric disorder group (CCS categories)	ICD10 codes
	T474X2S T475X2A T475X2D T475X2S T476X2A T476X2D T476X2S T477X2A T477X2D T477X2S T478X2A T478X2D T478X2S T4792XA T4792XD T4792XS T480X2A T480X2D T480X2S T481X2A T481X2D T481X2S T48202A T48202D T48202S T48292A T48292D T48292S T483X2A T483X2D T483X2S T484X2A T484X2D T484X2S T485X2A T485X2D T485X2S T486X2A T486X2D T486X2S T48902A T48902D T48902S T48992A T48992D T48992S T490X2A T490X2D T490X2S T491X2A T491X2D T491X2S T492X2A T492X2D T492X2S T493X2A T493X2D T493X2S T494X2A T494X2D T494X2S T495X2A T495X2D T495X2S T496X2A T496X2D T496X2S T497X2A T497X2D T497X2S T498X2A T498X2D T498X2S T4992XA T4992XD T4992XS T500X2A T500X2D T500X2S T501X2A T501X2D T501X2S T502X2A T502X2D T502X2S T503X2A T503X2D T503X2S T504X2A T504X2D T504X2S T505X2A T505X2D T505X2S T506X2A T506X2D T506X2S T507X2A T507X2D T507X2S T508X2A T508X2D T508X2S T50902A T50902D T50902S T50992A T50992D T50992S T50A12A T50A12D T50A12S T50A22A T50A22D T50A22S T50A92A T50A92D T50A92S T50B12A T50B12D T50B12S T50B92A T50B92D T50B92S T50Z12A T50Z12D T50Z12S T50Z92A T50Z92D T50Z92S T510X2A T510X2D T510X2S T511X2A T511X2D T511X2S T512X2A T512X2D T512X2S T513X2A T513X2D T513X2S T518X2A T518X2D T518X2S T5192XA T5192XD T5192XS T520X2A T520X2D T520X2S T521X2A T521X2D T521X2S T522X2A T522X2D T522X2S T523X2A T523X2D T523X2S T524X2A T524X2D T524X2S T528X2A T528X2D T528X2S T5292XA T5292XD T5292XS T530X2A T530X2D T530X2S T531X2A T531X2D T531X2S T532X2A T532X2D T532X2S T533X2A T533X2D T533X2S T534X2A T534X2D T534X2S T535X2A T535X2D T535X2S T536X2A T536X2D T536X2S T537X2A T537X2D T537X2S T5392XA T5392XD T5392XS T540X2A T540X2D T540X2S T541X2A T541X2D T541X2S T542X2A T542X2D T542X2S T543X2A T543X2D T543X2S T5492XA T5492XD T5492XS T550X2A T550X2D T550X2S T551X2A T551X2D T551X2S T560X2A T560X2D T560X2S T561X2A T561X2D T561X2S T562X2A T562X2D T562X2S T563X2A T563X2D T563X2S T564X2A T564X2D T564X2S T565X2A T565X2D T565X2S T566X2A T566X2D T566X2S T567X2A T567X2D T567X2S T56812A T56812D T56812S T56892A T56892D T56892S T5692XA

© 2023 Singh P et al. *JAMA Network Open.*

Psychiatric disorder group (CCS categories)	ICD10 codes
	T5692XD T5692XS T570X2A T570X2D T570X2S T571X2A T571X2D T571X2S T572X2A T572X2D T572X2S T573X2A T573X2D T573X2S T578X2A T578X2D T578X2S T5792XA T5792XD T5792XS T5802XA T5802XD T5802XS T5812XA T5812XD T5812XS T582X2A T582X2D T582X2S T588X2A T588X2D T588X2S T5892XA T5892XD T5892XS T590X2A T590X2D T590X2S T591X2A T591X2D T591X2S T592X2A T592X2D T592X2S T593X2A T593X2D T593X2S T594X2A T594X2D T594X2S T595X2A T595X2D T595X2S T596X2A T596X2D T596X2S T597X2A T597X2D T597X2S T59812A T59812D T59812S T59892A T59892D T59892S T5992XA T5992XD T5992XS T600X2A T600X2D T600X2S T601X2A T601X2D T601X2S T602X2A T602X2D T602X2S T603X2A T603X2D T603X2S T604X2A T604X2D T604X2S T608X2A T608X2D T608X2S T6092XA T6092XD T6092XS T6102XA T6102XD T6102XS T6112XA T6112XD T6112XS T61772A T61772D T61772S T61782A T61782D T61782S T618X2A T618X2D T618X2S T6192XA T6192XD T6192XS T620X2A T620X2D T620X2S T621X2A T621X2D T621X2S T622X2A T622X2D T622X2S T628X2A T628X2D T628X2S T6292XA T6292XD T6292XS T63002A T63002D T63002S T63012A T63012D T63012S T63022A T63022D T63022S T63032A T63032D T63032S T63042A T63042D T63042S T63062A T63062D T63062S T63072A T63072D T63072S T63082A T63082D T63082S T63092A T63092D T63092S T63112A T63112D T63112S T63122A T63122D T63122S T63192A T63192D T63192S T632X2A T632X2D T632X2S T63302A T63302D T63302S T63312A T63312D T63312S T63322A T63322D T63322S T63332A T63332D T63332S T63392A T63392D T63392S T63412A T63412D T63412S T63422A T63422D T63422S T63432A T63432D T63432S T63442A T63442D T63442S T63452A T63452D T63452S T63462A T63462D T63462S T63482A T63482D T63482S T63512A T63512D T63512S T63592A T63592D T63592S T63612A T63612D T63612S T63622A T63622D T63622S T63632A T63632D T63632S T63692A T63692D T63692S T63712A T63712D T63712S T63792A T63792D T63792S T63812A T63812D T63812S T63822A T63822D T63822S T63832A T63832D T63832S T63892A T63892D T63892S T6392XA T6392XD T6392XS T6402XA T6402XD T6402XS T6482XA T6482XD T6482XS T650X2A T650X2D T650X2S T651X2A T651X2D T651X2S

© 2023 Singh P et al. *JAMA Network Open.*

Psychiatric disorder group (CCS categories)	ICD10 codes
	T65212A T65212D T65212S T65222A T65222D T65222S T65292A T65292D T65292S T653X2A T653X2D T653X2S T654X2A T654X2D T654X2S T655X2A T655X2D T655X2S T656X2A T656X2D T656X2S T65812A T65812D T65812S T65822A T65822D T65822S T65832A T65832D T65832S T65892A T65892D T65892S T6592XA T6592XD T6592XS T71112A T71112D T71112S T71122A T71122D T71122S T71132A T71132D T71132S T71152A T71152D T71152S T71162A T71162D T71162S T71192A T71192D T71192S T71222A T71222D T71222S T71232A T71232D T71232S X710XXA X710XXD X710XXS X711XXA X711XXD X711XXS X712XXA X712XXD X712XXS X713XXA X713XXD X713XXS X718XXA X718XXD X718XXS X719XXA X719XXD X719XXS X72XXXA X72XXXD X72XXXS X730XXA X730XXD X730XXS X731XXA X731XXD X731XXS X732XXA X732XXD X732XXS X738XXA X738XXD X738XXS X739XXA X739XXD X739XXS X7401XA X7401XD X7401XS X7402XA X7402XD X7402XS X7409XA X7409XD X7409XS X748XXA X748XXD X748XXS X749XXA X749XXD X749XXS X75XXXA X75XXXD X75XXXS X76XXXA X76XXXD X76XXXS X770XXA X770XXD X770XXS X771XXA X771XXD X771XXS X772XXA X772XXD X772XXS X773XXA X773XXD X773XXS X778XXA X778XXD X778XXS X779XXA X779XXD X779XXS X780XXA X780XXD X780XXS X781XXA X781XXD X781XXS X782XXA X782XXD X782XXS X788XXA X788XXD X788XXS X789XXA X789XXD X789XXS X79XXXA X79XXXD X79XXXS X80XXXA X80XXXD X80XXXS X810XXA X810XXD X810XXS X811XXA X811XXD X811XXS X818XXA X818XXD X818XXS X820XXA X820XXD X820XXS X821XXA X821XXD X821XXS X822XXA X822XXD X822XXS X828XXA X828XXD X828XXS X830XXA X830XXD X830XXS X831XXA X831XXD X831XXS X832XXA X832XXD X832XXS X838XXA X838XXD X838XXS Z915

© 2023 Singh P et al. *JAMA Network Open.*

eTable 2: Description of Study Data From UCHDW in 3-Month Groups, 2020 to Dec 2021

	Mean monthly ED visits for schizophrenia spectrum disorders (Standard Deviation)	Mean monthly ED visits for all other psychiatric conditions (Standard Deviation)
Jan-March 2020	516 (50.1)	4961 (417.8)
April-June 2020	568 (66.1)	4095.7 (454.1)
July-September 2020	581.3 (26.1)	4668 (18.7)
October-December 2020	553.8 (39.8)	4377 (442.5)
Jan-March 2021	514.3 (51.2)	4076.7 (296)
April-June 2021	515 (57.6)	4593.7 (306.9)
July-September 2021	464 (47.1)	4410.3 (238.5)
October-December 2021	501.3 (38.4)	4221.3 (134.2)

© 2023 Singh P et al. *JAMA Network Open.*

eFigure. Time-Series Graph of Autocorrelation Function (ACF) (Panel A) and Partial Autocorrelation Function (PACF) (Panel B) for ED Visits for Schizophrenia Spectrum Disorders Across 5 UC Campuses (Shown for First 24 Months)

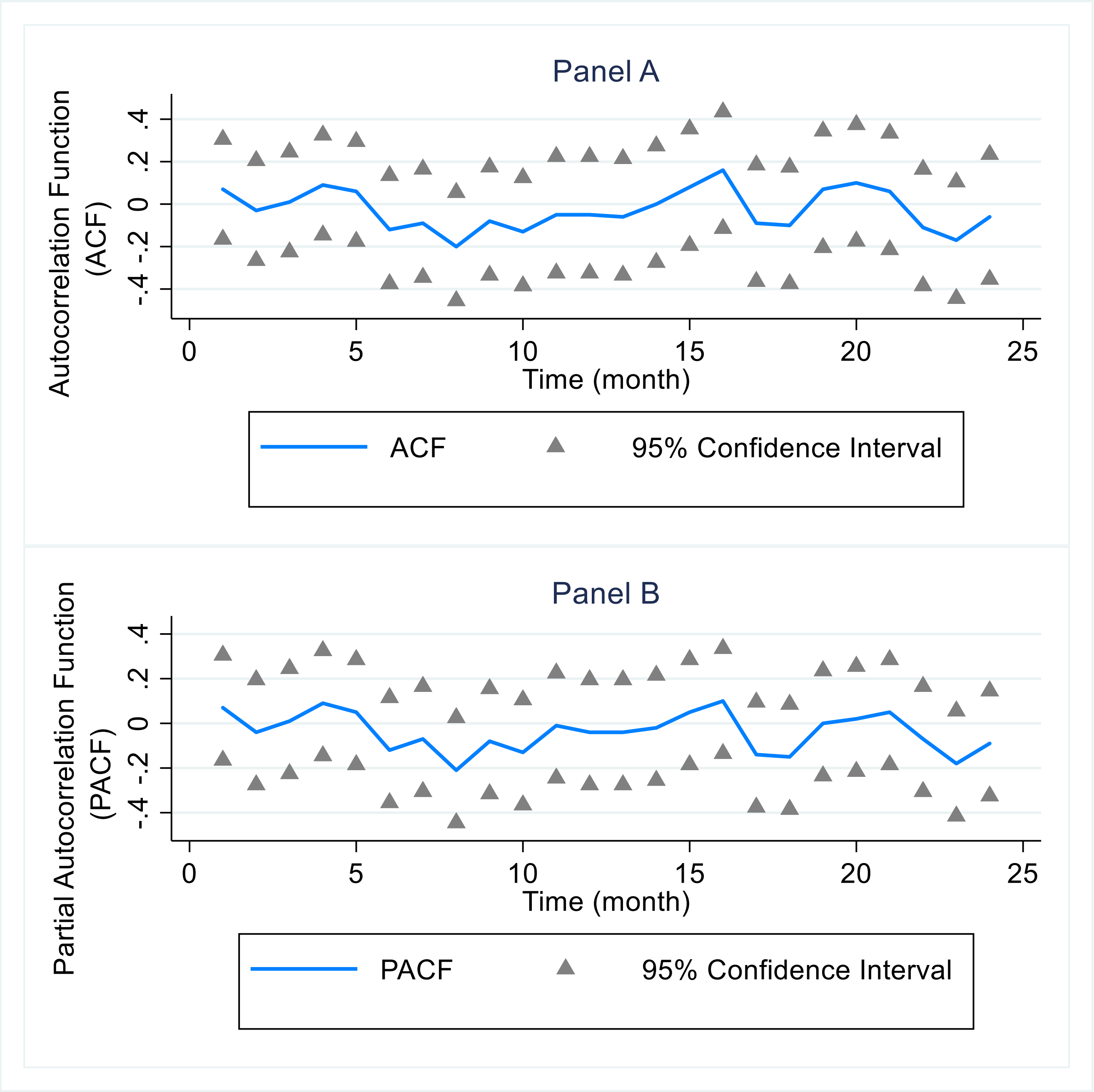

© 2023 Singh P et al. *JAMA Network Open.*

eTable 3: Time-Series Results Predicting Monthly Values of ED Visits for Schizophrenia Spectrum Disorders From January 2016 to December 2021 Across 5 UC Campuses
Monthly counts are modelled as a function of the initial phase of the COVID-19 pandemic (binary, March-May 2020), 3 months post initial phase of the COVID-19 pandemic (binary, June-August 2020), ED visits for all other psychiatric conditions, and autocorrelation.

**Variable**	**Coef.**	**SE**	**P value**
Constant	383.3	58	<0.001
ED visits for all other psychiatric conditions	0.03	0.01	0.018
Autoregressive Parameter (AR)			
AR 3	0.25	0.12	0.033
COVID-19 pandemic (initial phase, March-May 2020) (lag 0)	14.8	22.2	0.51
3 months post initial phase of the COVID-19 pandemic (June-August 2020) (lag 0)	81.3	20.6	<0.001

eTable 4: Time-Series Results Predicting Monthly Values of ED Visits for Schizophrenia Spectrum Disorders From January 2016 to December 2021 Across 2 UC Campuses in Northern California (UC Davis, UC San Francisco).
Monthly counts are modelled as a function of the initial phase of the COVID-19 pandemic (binary, March-May 2020), 3 months post initial phase of the COVID-19 pandemic (binary, June-August 2020), ED visits for all other psychiatric conditions, and autocorrelation.

**Variable**	**Coef.**	**SE**	**P value**
Constant	98.7	32.4	0.003
ED visits for all other psychiatric conditions	-0.03	0.03	0.17
Autoregressive Parameter (AR)			
AR 3	0.51	0.11	<0.001
COVID-19 pandemic (initial phase, March-May 2020) (lag 0)	32.4	14.6	0.03
3 months post initial phase of the COVID-19 pandemic (June-August 2020) (lag 0)	29.7	13.8	0.03

© 2023 Singh P et al. *JAMA Network Open.*

eTable 5: Time-Series Results Predicting Monthly Values of ED Visits for Schizophrenia Spectrum Disorders From January 2016 to December 2021 Across 3 UC Campuses in Southern California (UC Los Angeles, UC Irvine, UC San Diego). Monthly counts are modelled as a function of the initial phase of the COVID-19 pandemic (binary, March-May 2020), 3 months post initial phase of the COVID-19 pandemic (binary, June-August 2020), ED visits for all other psychiatric conditions, and autocorrelation.

**Variable**	**Coef.**	**SE**	**P value**
Constant	18.3	41.3	0.661
ED visits for all other psychiatric conditions	0.05	0.01	<0.001
Autoregressive Parameter (AR)			
AR 3	0.26	0.12	0.027
COVID-19 pandemic (initial phase, March-May 2020) (lag 0)	5.39	24.1	0.83
3 months post initial phase of the COVID-19 pandemic (June-August 2020) (lag 0)	95.96	22.2	<0.001

eTable 6: Time-Series Results Predicting Monthly Values of ED Visits for Schizophrenia Spectrum Disorders From January 2016 to December 2021 in 5 UC Campuses. Monthly counts are modelled as a function of the initial phase of the COVID-19 pandemic (binary, March-May 2020, 0 to 3 month lags), and autocorrelation. Analyses do not control for ED visits for all other psychiatric conditions.

**Variable**	**Coef.**	**SE**	**P value**
Constant	523.6	6.02	<0.001
Autoregressive Parameter (AR)			
AR 3	0.26	0.12	0.034
COVID-19 pandemic (initial phase, March-May 2020)			
Lag 0	-34.4	27.5	0.215
Lag 1	48.8	31.2	0.121
Lag 2	-14.03	31.5	0.654
Lag 3	69.5	27.7	0.014

© 2023 Singh P et al. *JAMA Network Open.*

eTable 7: Time-Series Results Predicting Monthly Values of ED Visits for Schizophrenia Spectrum Disorders From January 2016 to December 2021 in 5 UC Campuses. Monthly counts are modelled as a function of the extended phase of the COVID-19 pandemic (binary, March-December 2020, 0 to 3 month lags), and autocorrelation. Analyses do not control for ED visits for all other psychiatric conditions.

Variable	Coef.	SE	P value
Constant	523.9	4.56	<0.001
Autoregressive Parameter (AR)			
AR 12	-0.3	0.14	0.037
COVID-19 pandemic (extended phase, March-December 2020)			
Lag 0	-24.5	26.8	0.366
Lag 1	84.8	37.1	0.026
Lag 2	-29.9	36.2	0.409
Lag 3	5.2	26.4	0.842

eTable 8: Time-Series Results Predicting Monthly Values of ED Visits for All Other Psychiatric Conditions From January 2016 to December 2021 in 5 UC Campuses. Monthly counts are modelled as a function of the initial phase of the COVID-19 pandemic (binary, March-May 2020, 0 to 3 month lags), and autocorrelation.

Variable	Coef.	SE	P value
Constant	5102.9	328.2	<0.001
Autoregressive Parameter (AR)			
AR 1	0.77	0.09	<0.001
AR 12	0.54	0.12	<0.001
COVID-19 pandemic (initial phase, March-May 2020)			
Lag 0	-804.9	215.6	<0.001
Lag 1	-393.4	183.7	0.036
Lag 2	86.3	182.8	0.640
Lag 3	-192	208.9	0.361

© 2023 Singh P et al. *JAMA Network Open.*

eTable 9: Time-Series Results Predicting Monthly Values of ED Visits for All Other Psychiatric Conditions From January 2016 to December 2021 in 5 UC Campuses. Monthly counts are modelled as a function of the extended phase of the COVID-19 pandemic (binary, March-December 2020, 0 to 3 month lags), and autocorrelation.

Variable	Coef.	SE	P value
Constant	5166.5	257.6	<0.001
Autoregressive Parameter (AR)			
AR 1	0.73	0.10	<0.001
AR 12	0.45	0.13	0.001
COVID-19 pandemic (extended phase, March-December 2020)			
Lag 0	-360.7	221.8	0.107
Lag 1	-377.3	215.2	0.084
Lag 2	-15.3	210.8	0.944
Lag 3	197.9	208.4	0.345

© 2023 Singh P et al. *JAMA Network Open.*
