# Supplementary material for: ED Visits for Schizophrenia Spectrum Disorders During the COVID-19 Pandemic at 5 Campus Health Systems
Source: JAMA Netw Open. 2023 Dec 27;6(12):e2349305. doi: 10.1001/jamanetworkopen.2023.49305 (PMC10753394; doi:10.1001/jamanetworkopen.2023.49305)
Supplement: Supplement 2. — Data Sharing Statement [file jamanetwopen-e2349305-s002.pdf]

Data Sharing Statement

Singh. ED Visits for Schizophrenia Spectrum Disorders During the COVID-19 Pandemic at 5 Campus Health Systems. *JAMA Netw Open*. Published December 27, 2023. doi:10.1001/jamanetworkopen.2023.49305

Data
Data available: No

Additional Information
Explanation for why data not available: The UCHDW does not allow Authors to share data. Researchers can submit a request directly to the UCHDW for data access.
